# Governance of artificial intelligence for health systems, WHO European Region

**DOI:** 10.2471/BLT.25.294978

**Published:** 2026-06-01

**Authors:** Keyrellous Adib, Nicholas Letchford, Hanna Emilia Dunning, Nagui Salama, Yiannos Tolias, Jerome De Barros, Natasha Azzopardi-Muscat, Hans Henri P Kluge, David Novillo-Ortiz

**Affiliations:** a World Health Organization Regional Office for Europe, UN City, Marmorvej 51, 2100 Copenhagen, Denmark.; b European Commission, Brussels, Belgium.

## Abstract

**Objective:**

To provide an overview of the status of artificial intelligence (AI) governance for health in the World Health Organization (WHO) European Region.

**Methods:**

The WHO Regional Office for Europe developed a cross-sectional survey on AI governance and adoption. The survey was available to all 53 Member States between June 2024 and March 2025. Responses were analysed at the regional and subregional level.

**Findings:**

Of the 50 Member States responding to the survey, 8% (4/50) have a health-specific AI strategy and 14% (7) are developing one. Regionally, 33 Member States have implemented cross-sectoral AI strategies and 8 (16%) are developing them. Of these Member States, almost half each have assigned implementation and oversight responsibilities to existing single or multiple government agencies. Nearly half the Member States (23) reported ongoing assessments of laws and policies on AI systems and a fifth (10) have developed new health-specific AI laws. Only 14 Member States have issued guidelines to address the ethical implications of using AI in health or across sectors. Less than 10% (4) of Member States have developed liability standards for AI or guidance on the application of existing liability standards. Overall, 33 (66%) Member States have adopted health data strategies, 33 (66%) have health data hubs and (68%) have health data authorities supporting data access and control.

**Conclusion:**

In the WHO European Region, governance of AI in health care is underdeveloped. Adaptive legal and policy mechanisms are needed to respond effectively to the complex and evolving challenges of AI integration in health systems.

## Résumé

**Objectif:**

Offrir un aperçu de l’état de la gouvernance de l’intelligence artificielle (IA) dans le domaine de la santé au sein de la région Europe de l’Organisation mondiale de la Santé (OMS).

**Méthodes:**

Le Bureau régional de l’OMS pour l’Europe a élaboré une enquête transversale sur la gouvernance et l’adoption de l’IA. Cette enquête a été mise à la disposition des 53 États membres entre juin 2024 et mars 2025. Les réponses ont été analysées aux niveaux régional et sous-régional.

**Résultats:**

Sur les 50 États membres ayant répondu à l’enquête, 8% (4/50) disposent d’une stratégie d’IA spécifique au domaine de la santé et 14% (7) en sont en train d’élaborer une. À l’échelle régionale, 33 États membres ont mis en œuvre des stratégies intersectorielles en matière d’IA et 8 (16%) sont en train de les élaborer. Parmi ces États membres, près de la moitié ont confié les responsabilités de mise en œuvre et de surveillance à une ou plusieurs agences gouvernementales existantes. Près de la moitié des États membres (23) ont indiqué mener actuellement des évaluations des lois et politiques relatives aux systèmes d’IA, et un cinquième (10) ont élaboré de nouvelles lois sur l’IA qui sont spécifiques au domaine de la santé. Seuls 14 États membres ont publié des lignes directrices visant à traiter les implications éthiques de l’utilisation de l’IA dans le domaine de la santé ou dans d’autres secteurs. Moins de 10% (4) des États membres ont mis au point des normes de responsabilité pour l’IA ou des orientations sur l’application de normes de responsabilité existantes. Dans l’ensemble, 33 États membres (66%) ont adopté des stratégies en matière de données de santé, 33 (66%) disposent de plateformes de données de santé et 68% ont mis en place des autorités chargées des données de santé qui facilitent l’accès aux données et leur régulation.

**Conclusion:**

Dans la région Europe de l’OMS, la gouvernance de l’IA dans les soins de santé est insuffisamment développée. Des mécanismes juridiques et politiques adaptatifs sont nécessaires pour répondre efficacement aux défis complexes et en constante évolution liés à l’intégration de l’IA dans les systèmes de santé.

## Resumen

**Objetivo:**

Proporcionar una visión general del estado de la gobernanza de la inteligencia artificial (IA) para la salud en la Región Europea de la Organización Mundial de la Salud (OMS).

**Métodos:**

La Oficina Regional de la OMS para Europa desarrolló una encuesta transversal sobre la gobernanza y la adopción de la IA. La encuesta estuvo disponible para los 53 Estados Miembros entre junio de 2024 y marzo de 2025. Las respuestas se analizaron a nivel regional y subregional.

**Resultados:**

De los 50 Estados Miembros que respondieron a la encuesta, el 8% (4/50) cuenta con una estrategia de IA específica para la salud y el 14% (7) se encuentra desarrollando una. A nivel regional, 33 Estados Miembros han implementado estrategias de IA intersectoriales y 8 (16%) se encuentran en proceso de desarrollarlas. De estos Estados Miembros, casi la mitad han asignado responsabilidades de implementación y supervisión a una o varias agencias gubernamentales existentes. Casi la mitad de los Estados Miembros (23) informaron de evaluaciones en curso de leyes y políticas sobre sistemas de IA, y una quinta parte (10) ha desarrollado nuevas leyes de IA específicas para la salud. Solo 14 Estados Miembros han emitido directrices para abordar las implicaciones éticas del uso de la IA en la salud o en distintos sectores. Menos del 10% (4) de los Estados Miembros ha desarrollado normas de responsabilidad para la IA o directrices sobre la aplicación de normas de responsabilidad existentes. En conjunto, 33 (66%) Estados Miembros han adoptado estrategias de datos sanitarios, 33 (66%) disponen de repositorios de datos sanitarios y el 68% cuenta con autoridades de datos sanitarios que apoyan el acceso y el control de los datos.

**Conclusión:**

En la Región Europea de la OMS, la gobernanza de la IA en la asistencia sanitaria está poco desarrollada. Se requieren mecanismos jurídicos y de política adaptativos para responder eficazmente a los desafíos complejos y cambiantes de la integración de la IA en los sistemas sanitarios.

## ملخص

**الغرض:**

تقديم نظرة عامة عن حالة حوكمة الذكاء الاصطناعي (AI) في مجال الصحة في المنطقة الأوروبية لمنظمة الصحة العالمية (WHO).

**الطريقة:**

أعد المكتب الإقليمي لمنظمة الصحة العالمية لأوروبا مسحًا يشمل عدة قطاعات حول حوكمة الذكاء الاصطناعي وتطبيقه. وأتيح المسح لجميع الدول الأعضاء البالغ عددها 53 دولة في الفترة ما بين يونيو/حزيران 2024، ومارس/آذار 2025. تم تحليل الردود على المستويين الإقليمي وتحت الإقليمي.

**النتائج:**

من بين الدول الأعضاء الـ 50 التي شاركت في المسح، كان لدى %8 (4/50) منها استراتيجية خاصة بالذكاء الاصطناعي في مجال الصحة، وتسعى %14 (7) منها لوضع استراتيجية لها. على الصعيد الإقليمي، قامت 33 دولة عضو بتنفيذ استراتيجيات للذكاء الاصطناعي تشمل عدة قطاعات، وتقوم 8 دول (%16) لوضع استراتيجية. ومن بين هذه الدول الأعضاء، كلف ما يقرب من نصفها وكالة حكومية، قائمة واحدة أو أكثر، بمسؤوليات التنفيذ والرقابة. وأفاد ما يقرب من نصف الدول الأعضاء (23) بإجراء تقييمات مستمرة للقوانين والسياسات المتعلقة بأنظمة الذكاء الاصطناعي، ووضع خُمسها (10) قوانين جديدة خاصة بالذكاء الاصطناعي في مجال الصحة. ولم تصدر سوى 14 دولة عضو مبادئ توجيهية لمواجهة الآثار الأخلاقية لاستخدام الذكاء الاصطناعي في مجال الصحة أو عبر القطاعات. ووضعت أقل من %10 (4) من الدول الأعضاء معايير للمسؤولية فيما يتعلق بالذكاء الاصطناعي أو إرشادات بشأن تطبيق معايير المسؤولية القائمة. بوجه عام، اعتمدت 33 دولة عضو (%66) استراتيجيات للبيانات الصحية، ولدى 33 دولة عضو (%66) مراكز للبيانات الصحية، ولدى %68 منها هيئات معنية بالبيانات الصحية تدعم الوصول إلى البيانات ومراقبتها.

**الاستنتاج:**

في المنطقة الأوروبية لمنظمة الصحة العالمية، لا تزال حوكمة الذكاء الاصطناعي في مجال الرعاية الصحية في مرحلة متأخرة. وهناك حاجة إلى آليات قانونية وسياسية قابلة للتكيف من أجل الاستجابة بفعالية للتحديات المعقدة والمتغيرة التي ينطوي عليها دمج الذكاء الاصطناعي في النظم الصحية.

## 摘要

**目的:**

旨在概述世卫组织 (WHO) 欧洲区域在卫生领域的人工智能 (AI) 治理现状。

**方法:**

世卫组织欧洲区域办事处就 AI 治理和采用问题开展了一项横断面研究。在 2024 年 6 月至 2025 年 3 月期间,该项研究向 53 个会员国全面开放。我们在区域和次区域层面对研究结果进行了分析。

**结果:**

在参与该项研究的 50 个会员国中,有 8% (4/50) 已实施了针对卫生领域的 AI 战略,另有 14%(7 个)正处于战略制定阶段。在区域层面,有 33 个会员国已实施了跨部门 AI 战略,另有 8 个(占 16%)正处于战略制定阶段。在这些会员国中,已将实施职责和监督职责分别分配给一个或多个既有政府机构的会员国几乎各占一半。有近一半会员国(23 个)称其对 AI 系统相关法律和政策进行了持续评估,另有五分之一会员国(10 个)已制定了新的针对卫生领域的 AI 法律。仅 14 个会员国发布了旨在应对在卫生部门或跨部门使用 AI 所涉道德问题的指南。不到 10%(4 个)的会员国已制定了针对 AI 的责任标准或发布了有关如何应用现有责任标准的指南。总体而言,有 33 个会员国(占 66%)推行了卫生数据相关战略;有 33 个会员国(占 66%)建立了卫生数据中心;以及有 68% 的会员国成立了提供数据获取与控制服务的卫生数据管理机构。

**结论:**

世卫组织欧洲区域在卫生保健领域的 AI 治理框架尚不完善。需要构建自适应法律和政策机制,以有效应对在将 AI 集成至卫生系统过程中面临的不断演变的复杂挑战。

## Резюме

**Цель:**

Дать обзор состояния управления применением искусственного интеллекта (ИИ) для целей здравоохранения в Европейском регионе Всемирной организации здравоохранения (ВОЗ).

**Методы:**

Европейское региональное бюро ВОЗ разработало опрос в рамках поперечного исследования по вопросам управления в сфере ИИ и его внедрения. Опрос проводился среди всех 53 государств-членов в период с июня 2024 года по март 2025 года. Ответы были проанализированы на региональном и субрегиональном уровнях.

**Результаты:**

Из 50 государств-членов, принявших участие в опросе, 8% (4 из 50) имеют профильную стратегию ИИ в сфере здравоохранения и 14% (7) разрабатывают ее. На региональном уровне 33 государства-члена внедрили межсекторальные стратегии ИИ и 8 государств (16%) разрабатывают их. Почти в половине этих государств-членов обязанности по внедрению и надзору возложены на одно существующее ведомство, а в другой половине – на несколько. Почти половина государств-членов (23) сообщили о проведении оценки действующего законодательства и мер политики в отношении систем ИИ, и пятая часть (10) разработала новое законодательство относительно ИИ специально для сферы здравоохранения. Только 14 государств-членов разработали рекомендации по решению этических аспектов использования ИИ в здравоохранении или различных секторах. Менее 10% (4) государств-членов разработали стандарты юридической ответственности в сфере ИИ или рекомендации по применению имеющихся стандартов юридической ответственности. В целом 33 государства-члена (66%) внедрили стратегии относительно данных в здравоохранении, 33 (66%) имеют центры хранения и обработки данных по здравоохранению, в 68% созданы профильные органы, обеспечивающие поддержку доступа к медицинским данным и контроль над ними.

**Вывод:**

В Европейском регионе ВОЗ система управления применением ИИ в здравоохранении развита недостаточно. Необходимы адаптивные правовые и политические механизмы для эффективного реагирования на сложные и постоянно меняющиеся задачи, связанные с интеграцией ИИ в системы здравоохранения.

## Introduction

A robust governance ecosystem encompasses relevant strategies, laws, liability frameworks and regulations to ensure human safety and institutional accountability.^1^ Governance of artificial intelligence (AI) has several key dimensions. AI governance can be vertical and focused on specific sectors or domains, or horizontal, spanning multiple domains with cross-cutting rules. AI governance can be also centralized under a single governing authority or decentralized and involve multiple actors. Additionally, the legal framework can have different levels of enforceability, ranging from hard (binding legal instruments), soft (non-binding guidelines rather than standards) and so-called supersoft instruments such as the World Health Organization’s (WHO) ethical principles for the use of AI in health.^2^^,^^3^ AI governance also involves both private and public actors. Private regulation often overlaps with soft law through industry-led norms, codes of conduct and best practices, while public authorities contribute through legal instruments such as liability laws and regulatory oversight.^2^

Global, regional and national strategies play an important role in shaping the responsible development and deployment of AI in health. At the global level, frameworks such as WHO’s Global Strategy on Digital Health provide overarching guidance for using digital technologies, including AI.^4^ Regional strategies, including the digital health action plan of the WHO European Region,^5^ build on the goals of the global strategy by addressing shared priorities, such as integrating big data and AI into health systems, through defined objectives and illustrative actions (activities Member States and WHO are committed to doing). National strategies vary in structure and ambition, ranging from dedicated health-specific AI strategies to components within broader digital or cross-sectoral frameworks. Additionally, oversight mechanisms differ across countries and can be the responsibility of government agencies, expert councils or independent bodies.^6^

In parallel, legislation on AI is progressing through three main pathways: adapting existing laws; incorporating AI-specific provisions into current legal frameworks; and creating new laws. Many governments reinterpret traditional legal principles to address emerging AI risks, while others use a modular approach, adding AI clauses to existing regulations.^2^ Some jurisdictions are developing sector-specific legislation, while others cover multiple sectors.^2^ A leading example of the latter is the European Union (EU) AI Act,^7^ which aims to establish a coherent, risk-based framework using four risk categories: unacceptable, high, limited and low. This framework is particularly relevant to health care, where AI presents challenges that may affect fundamental rights and patient safety.^7^

AI governance operates within complex digital health and data ecosystems such as national or interconnected electronic health records, which 87% (45/52) of Member States of the WHO European Region are implementing.^8^ However, challenges remain. Some countries experience so-called data deserts, with unreliable health data systems and political or social constraints on data collection.^9^^–^^11^ Health information systems face fragmentation, interoperability challenges, a shortage of skilled professionals, and legal uncertainties on data access and secondary use.^12^ Despite existing challenges, the development of data hubs, which are platforms that mobilize large volumes and different types of health data and apply computing power to run complex research algorithms, is a positive step forward.^3^^,^^13^ In many Member States of the WHO European Region, these efforts support participation in the European Health Data Space, which aims to standardize health-care data, promote interoperability, empower patients and enable secure cross-border data exchange for care and research.^3^^,^^14^

Governing AI in health care is complex, particularly within different digital health and data ecosystems. This study aimed to understand current practices in AI governance for health by surveying Member States of the WHO European Region.

## Methods

### Study design

A technical team at the WHO Regional Office for Europe developed and administered a survey which was launched in June 2024 and remained open until March 2025.

The survey questionnaire included 60 items covering the core elements of AI for health (available in the online repository).^15^ The questionnaire combined closed-ended checkboxes with open text fields to capture details, explanations and relevant links. The structure and style were similar to that of the regional digital health survey,^12^ while avoiding duplication of the questions. WHO and EU colleagues reviewed the survey questions and response options to ensure alignment with WHO guidance and relevant current and forthcoming EU regulations, and to reflect different models of AI governance and adoption.

The survey was made available on a secure digital platform. In instances where direct online submission was not feasible, we collected responses using a structured Word document (Microsoft, Redmond, United States of America), which was subsequently included on the digital platform. We provided all survey instructions and questions in English and Russian.

### Participants

All 53 Member States of the WHO European Region were invited to participate in the survey. Each Member State nominated a national survey coordinator to oversee completion of the survey and identify and engage relevant national experts in AI for health. The number and expertise of contributors varied by country, and the responses reflected collaborative input from multiple stakeholders.

### Data processing

The WHO team compiled the responses submitted through both the online platform and Word document into a unified database, and reviewed the data set for consistency and completeness. Where needed, the WHO team contacted national coordinators to clarify unclear or inconsistent entries.

Some questions were dependent on Member States’ responses to earlier questions. In these instances, Member States that did not respond positively to the initial question were excluded from the number of respondents on which percentages were calculated.

### Analysis

The WHO team analysed the data descriptively to characterize the strategic and regulatory system for AI in health care across the WHO European Region. The focus was on understanding countries’ current position rather than performing statistical comparisons and no inferential analyses were done.

We report both the percentage of respondents answering each specific question and the number of responses received for that question. We analysed the data at both the regional level and by subregional groupings based on the geographic divisions defined by the United Nations Statistics Division: eastern (Belarus, Bulgaria, Czechia, Hungary, Poland, Republic of Moldova, Romania, Russian Federation, Slovakia and Ukraine); northern (Denmark, Estonia, Finland, Iceland, Ireland, Latvia, Lithuania, Norway, Sweden and United Kingdom of Great Britain and Northern Ireland); southern (Albania, Andorra, Croatia, Greece, Italy, Malta, Montenegro, North Macedonia, Portugal, San Marino, Serbia, Slovenia and Spain); and western (Austria, Belgium, France, Germany, Luxembourg, Netherlands (Kingdom of the) and Switzerland) Europe; and central (Kazakhstan, Kyrgyzstan, Tajikistan and Uzbekistan) and western (Armenia, Azerbaijan, Cyprus, Georgia, Israel and Türkiye) Asia.^16^ Additionally, we disaggregated data for the 27 Member States of the EU. We used Microsoft Excel and Python (Python Software Foundation, Wilmington, USA) for all analyses.

## Results

**Fig. 1:**
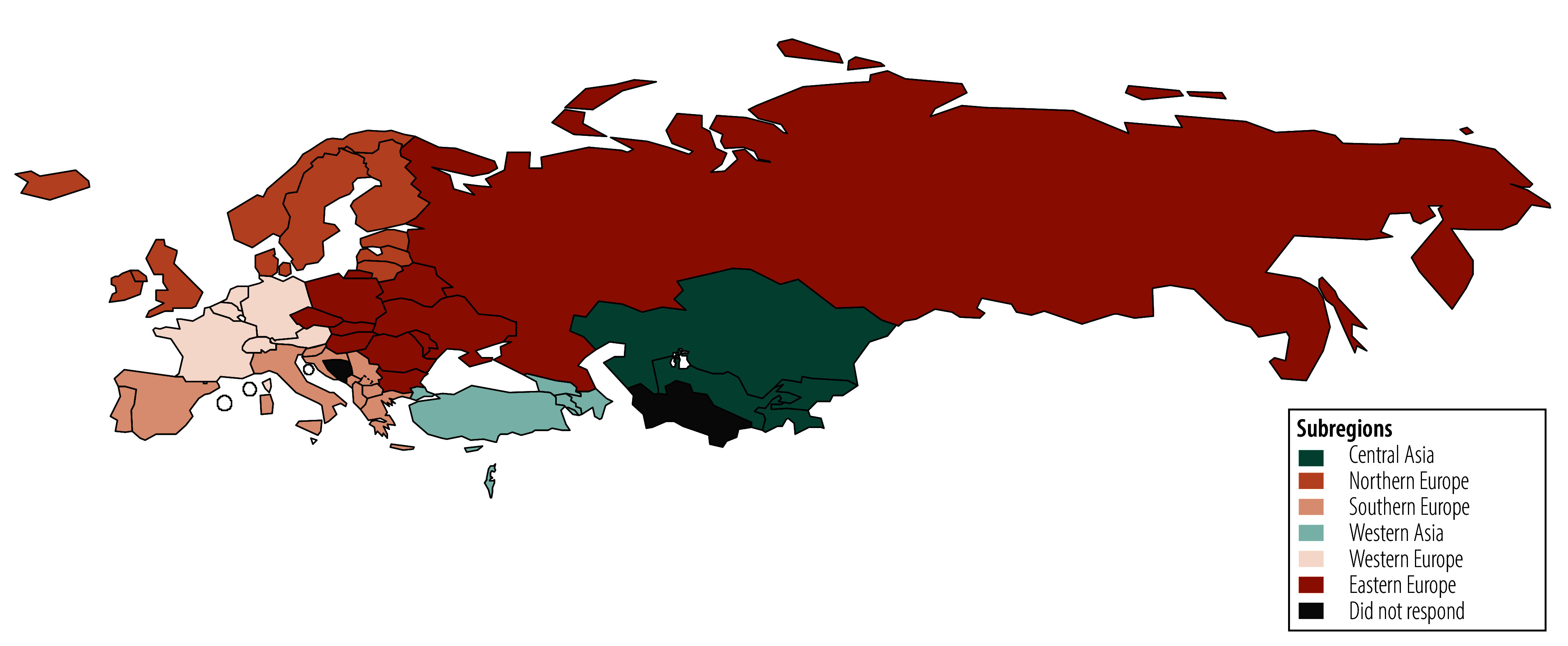
Member States of the WHO European Region, by subregion

Out of the 53 Member States of the WHO European Region invited to respond, 50 submitted responses, a response rate of 94%. Bosnia and Herzegovina, Monaco and Turkmenistan did not respond and were excluded from the analysis (Fig. 1).

WHO: World Health Organization.

Central Asia: Kazakhstan, Kyrgyzstan, Tajikistan, Uzbekistan; western Asia: Armenia, Azerbaijan, Cyprus, Georgia, Israel, Türkiye; eastern Europe: Belarus, Bulgaria, Czechia, Hungary, Poland, Republic of Moldova, Romania, Russian Federation, Slovakia, Ukraine; northern Europe: Denmark, Estonia, Finland, Iceland, Ireland, Latvia, Lithuania, Norway, Sweden, United Kingdom of Great Britain and Northern Ireland; southern Europe: Albania, Andorra, Croatia, Greece, Italy, Malta, Montenegro, North Macedonia, Portugal, San Marino, Serbia, Slovenia, Spain; western Europe: Austria, Belgium, France, Germany, Luxembourg, Netherlands (Kingdom of the), Switzerland. Did not respond: Bosnia and Herzegovina, Monaco, Turkmenistan. For the western Asia subregion, only countries that are part of the WHO European Region were considered. Western Asian countries in the WHO Eastern Mediterranean Region were excluded.

### Strategy models

**Fig. 2:**
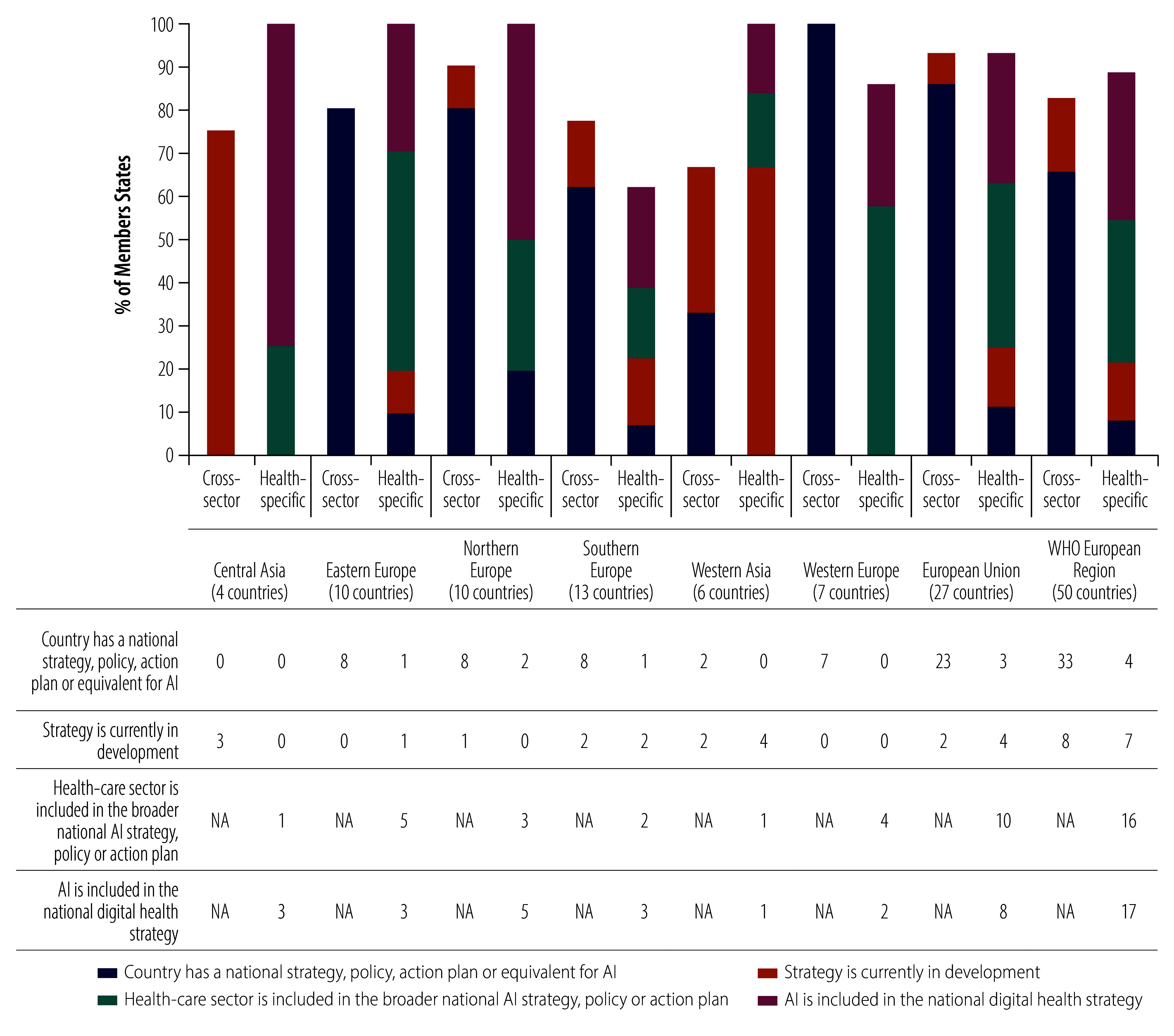
Presence of national strategies on AI, by region and subregion and type of strategy, WHO European Region

AI: artificial intelligence; WHO: World Health Organization.

Note: of the 53 Member States in the Region, 50 responded to the survey.

Member States have adopted different approaches to developing national strategies for AI in health (Fig. 2). Only 8% (4/50) of Member States have a specific national health AI strategy, while 66% (33/50) have a cross-sector AI strategy (list of strategies available in the online repository).^15^

All Member States in western Europe and about 80% (8/10) in eastern and northern Europe have a cross-sector AI strategy. In contrast, none of the European Region Member States in the central Asia area reported having cross-sector AI strategies; however, three are currently developing these strategies.

### Oversight of AI

**Fig. 3:**
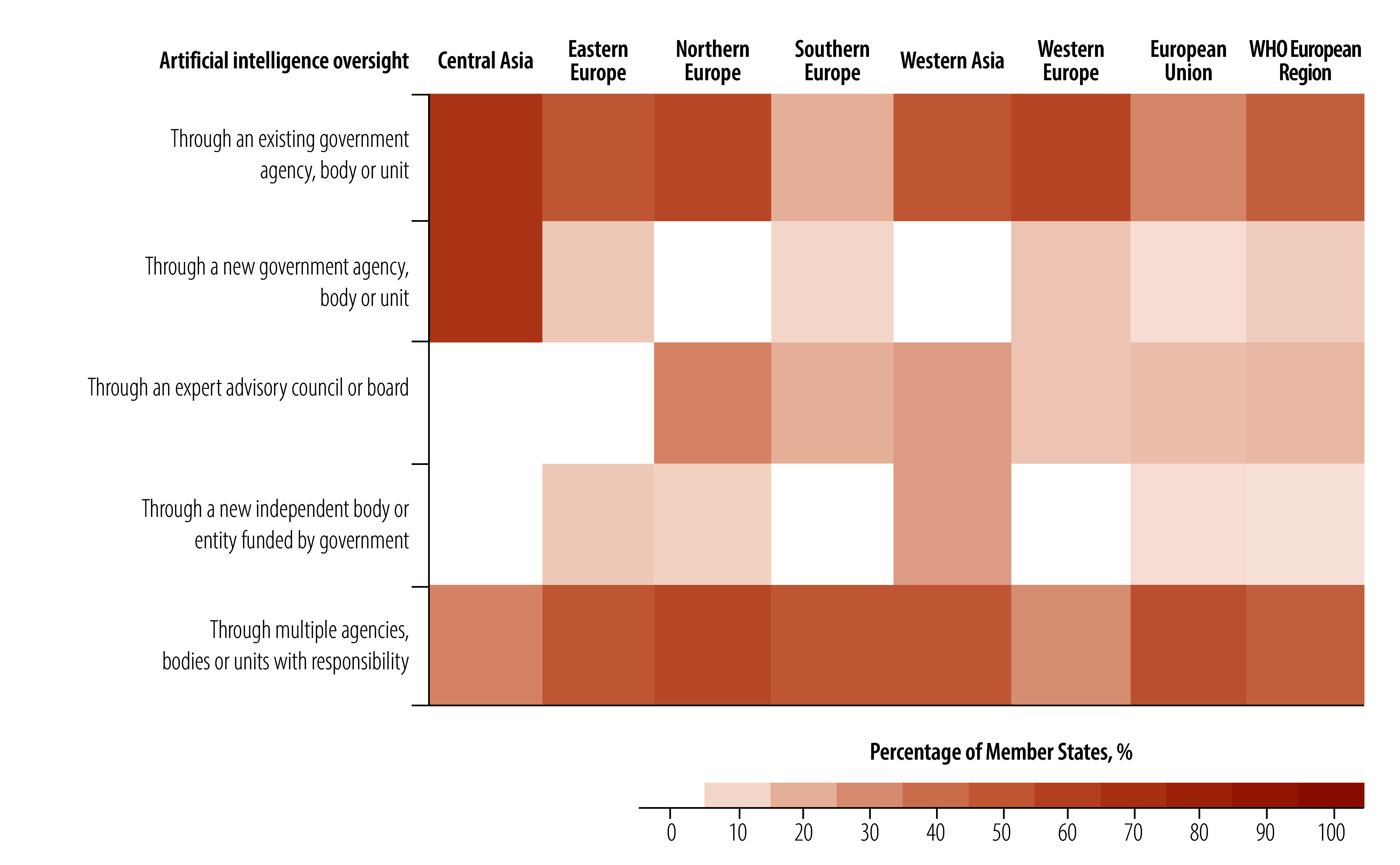
Oversight of implementation and operation of national AI initiatives in the health sector

Of the 41 Member States in the WHO European Region with established or developing cross-sector AI strategies, the two most common oversight approaches, each used by 46% (19/41) of Member States, assign oversight responsibilities either to an existing single government agency or to multiple government agencies (Fig. 3). Between 50% and 70% of Member States in northern Europe, western Europe and the central Asia area have oversight of AI assigned to an existing government agency. Except for central Asia Member States, establishing a new agency was the least common oversight approach among all subregions.

WHO: World Health Organization.

Notes: raw data are available in the online repository.^15^ Only countries reporting having or developing a national strategy, policy, action plan or equivalent for AI in the health sector is included.

**Fig. 4:**
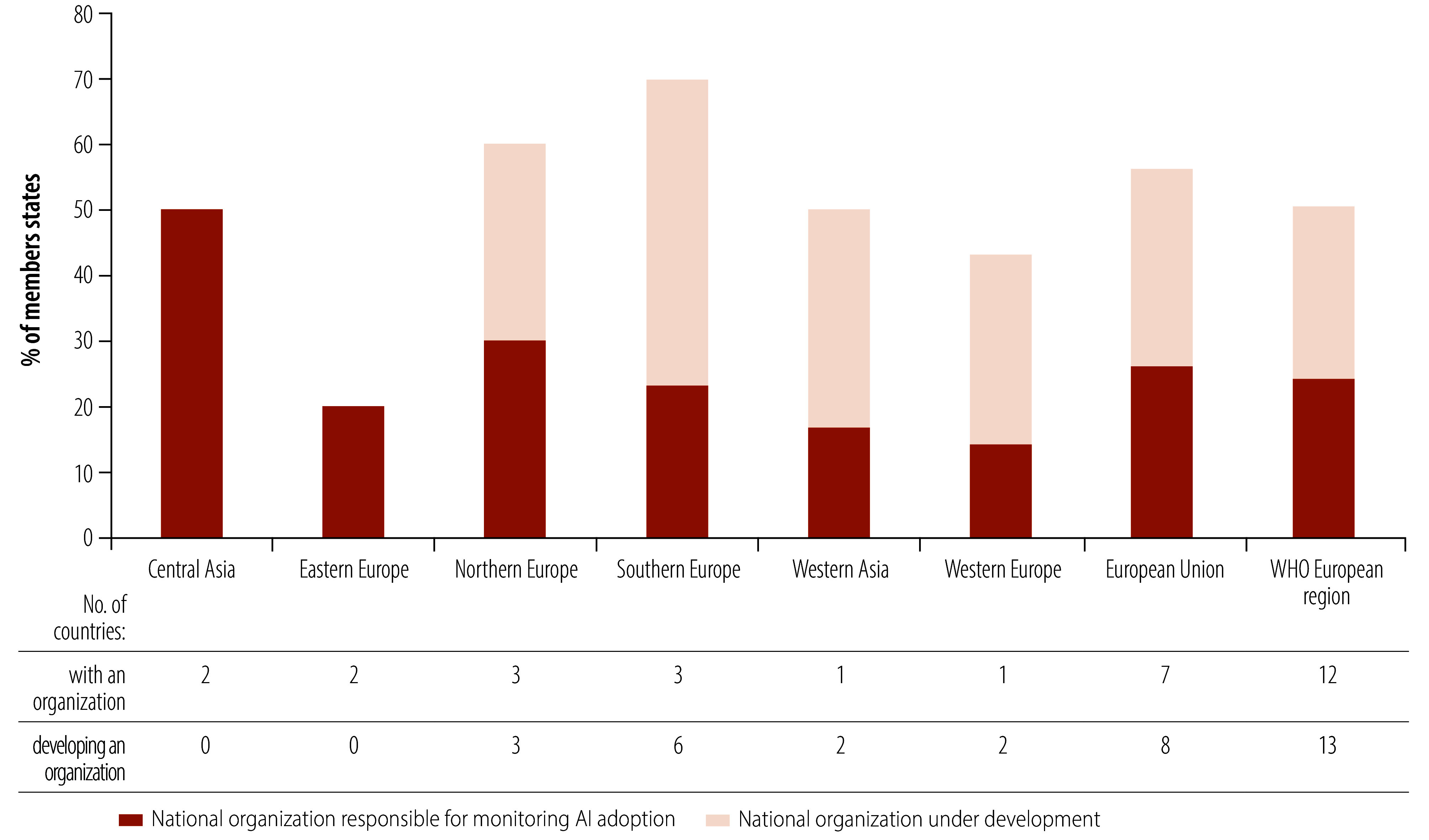
Presence of national organization responsible for monitoring AI adoption, by region and subregion, WHO European Region

AI: artificial intelligence; WHO: World Health Organization.

Note: of the 53 Member States in the Region, 50 responded to the survey.

Of the Member States in the European Region that responded, 24% (12/50) reported having a government agency currently responsible for monitoring AI use in health care, while 26% (13/50) indicated that such agencies are under development (Fig. 4). 

### Regulatory measures for AI

**Fig. 5:**
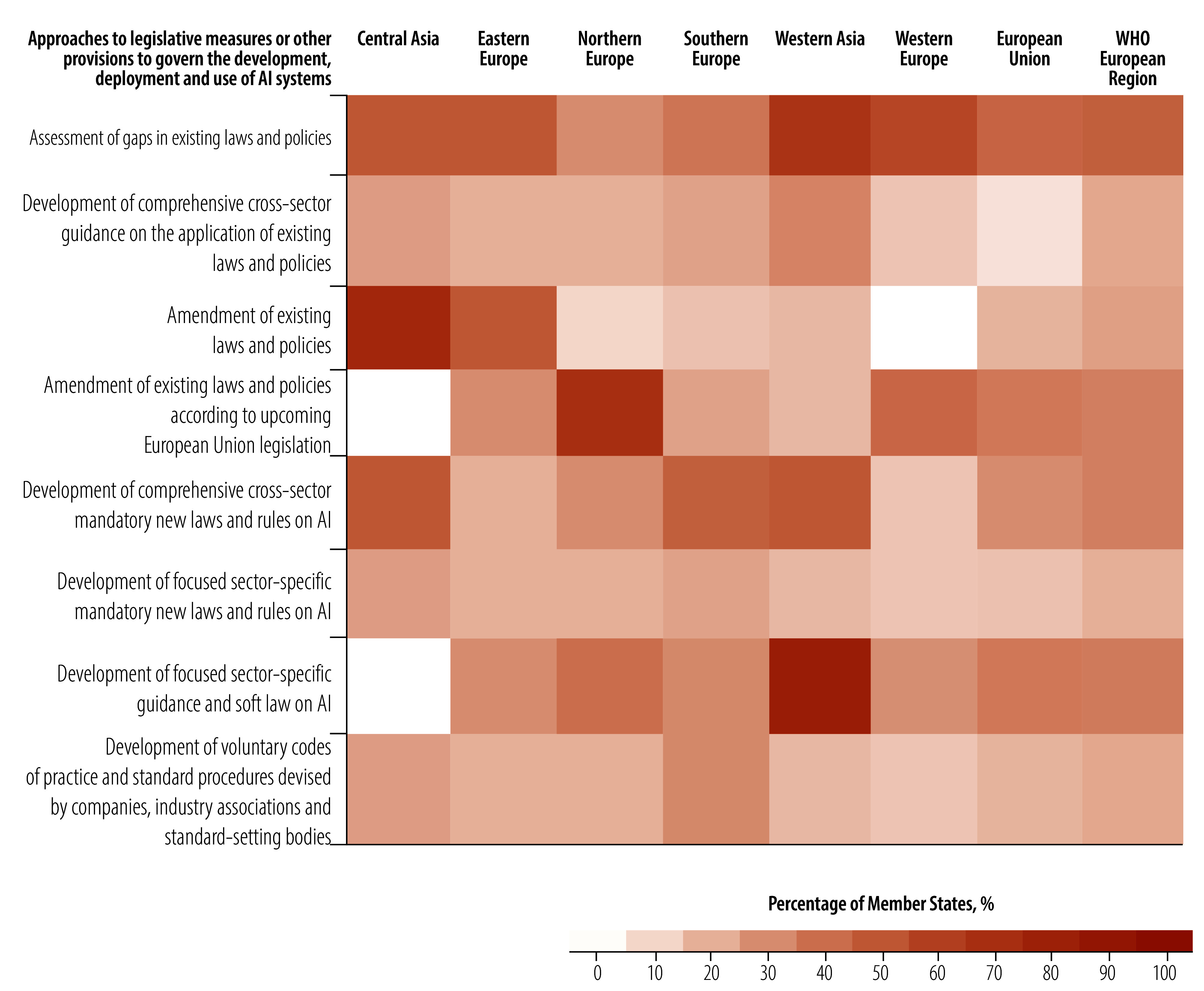
Regulatory approaches to governance of AI, by region and subregion, WHO European Region

AI: artificial intelligence; EU: European Union; WHO: World Health Organization.

Notes: raw data are available in the online repository.^15^ Of the 53 Member States in the Region, 50 responded to the survey.

As shown in Fig. 5, almost half of WHO European Region Member States assessed gaps in existing laws and policies that relate to AI systems. The least common approach was developing new health-specific laws for AI, a fifth (10) of Member States.

**Fig. 6:**
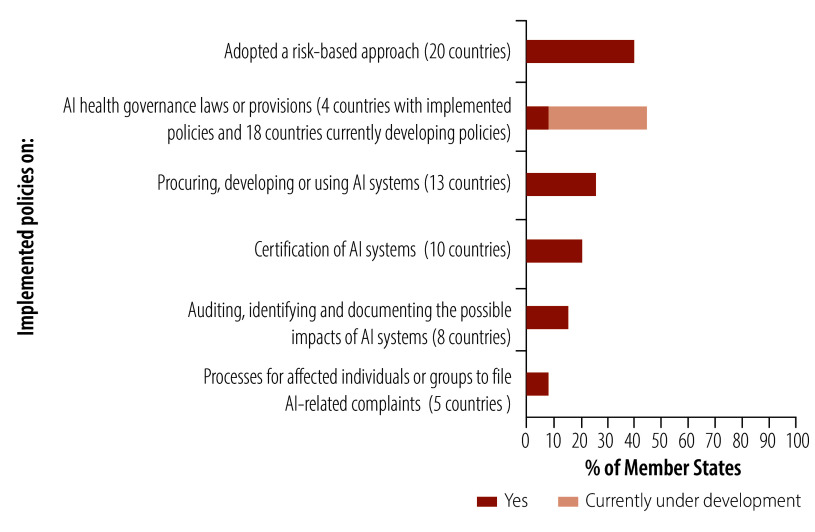
Focus of policies on AI implemented by Member States, WHO European Region

AI: artificial intelligence; WHO: World Health Organization.

Note: of the 53 Member States in the Region, 50 responded to the survey.

As regards policy, 26% (13/50) of Member States in the WHO European Region focused on policies related to procuring, developing and using AI systems in the health sector (Fig. 6). Only 10% (5/50) of Member States focused on processes for affected individuals to file AI-related complaints. Few Member States (4/50; 8%) have issued specific legislative measures or provisions for governance and oversight of AI in the health sector, with 36% (18/50) currently developing legislation.

Only 10% (5/50) of Member States have enacted three or more different types of AI-related health policies, with most of these Member States located in western and northern Europe. In contrast, 64% (32/50) of Member States reported having either no policies or being unsure of policies focused on AI in the health sector.

### Ethics and liability

**Fig. 7:**
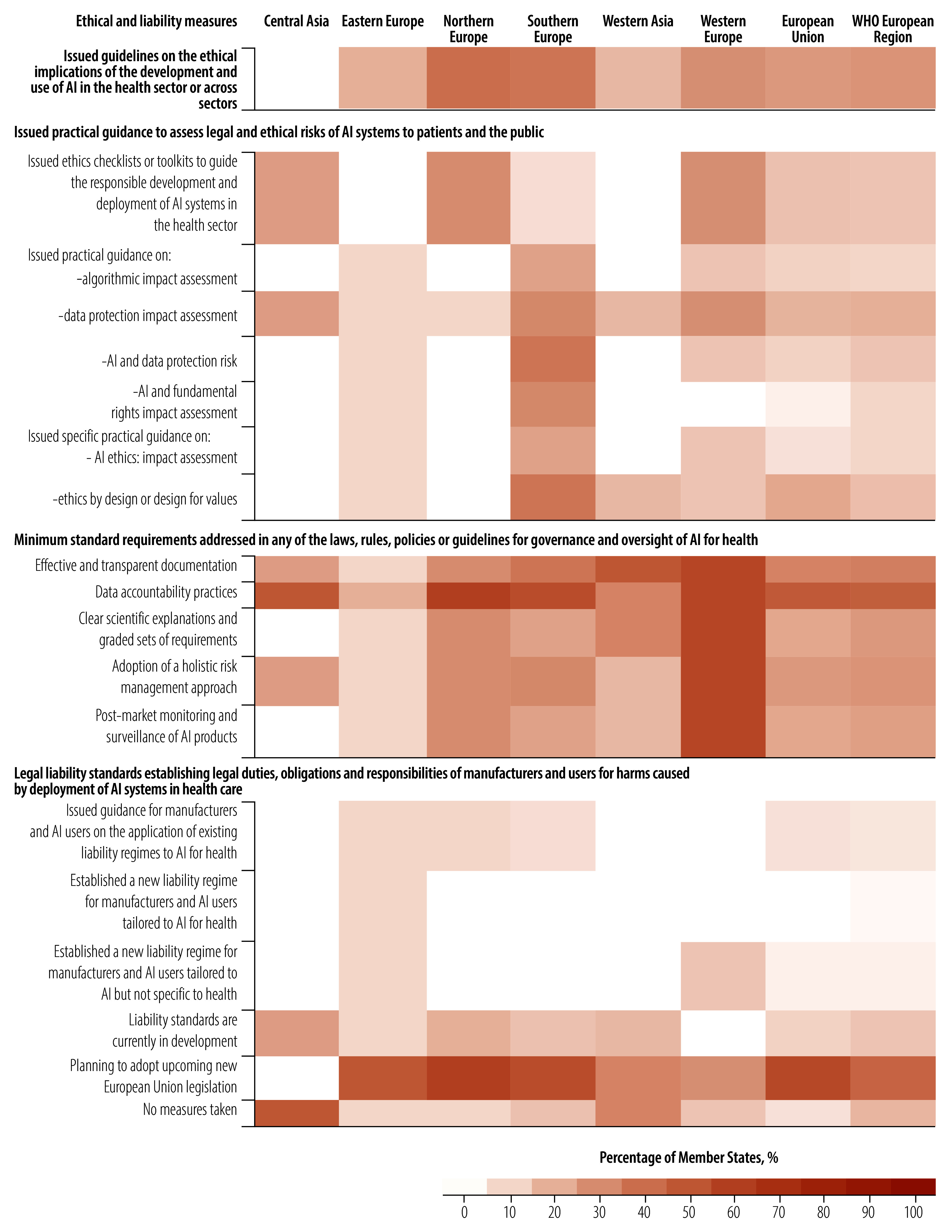
Ethical and liability measures taken on AI, by region and subregion, WHO European Region

AI: artificial intelligence; EU: European Union; WHO: World Health Organization.

Notes: raw data are available in the online repository.^15^ Of the 53 Member States in the Region, 50 responded to the survey.

**Fig. 8:**
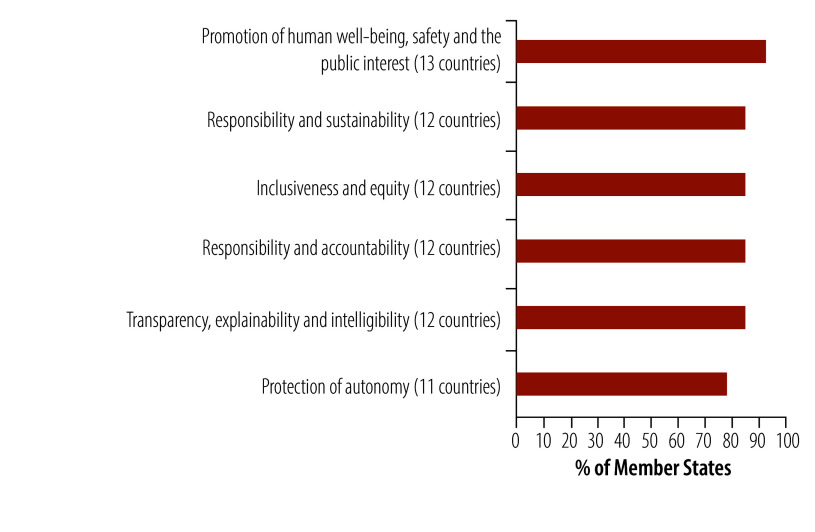
Ethical principles in guidelines on the development and use of artificial intelligence in the health sector by Member States, WHO European Region

Less than a third (28%; 14/50) of Member States have issued guidelines to address the ethical implications of using AI in health or across sectors (Fig. 7). Among these, only 8% (4/50) of Member States have issued health-specific AI guidelines to address ethical implications and 20% (10/50) have adopted cross-sectoral ethical frameworks. However, 38% (19/50) of Member States have yet to introduce any ethical guidance. The formulation of guidelines on the ethical implications of the development and use of AI was more common in Member States in northern and southern Europe (between 30% and 40% of Member States). Among the Member States that have issued health-specific or cross-sector ethical guidelines, 93% (13/14) address human well-being, safety and public interest (Fig. 8). 

WHO: World Health Organization.

Note: graph only includes the 14 countries reporting to have issued guidelines on the ethical implications of the development and use of AI in the health sector or across sectors.

Member States of the WHO European Region focused on a few potential legal and ethical risks to the public posed by AI systems: about 20% had issued practical guidance on data protection impact assessments and about 16% focused on ethics by design. More than half the Member States had issued no such guidance (Fig. 7). In the EU, 22% (6/27) of Member States had issued practical guidance on ethics by design.

The most common minimum standard requirement across the WHO European Region was to implement data accountability practices (almost half the Member States; 23/50). Less than 10% (4/50) of Member States had developed liability standards or guidance for manufacturers and users on the application of existing liability standards (Fig. 7).

### Data governance for secondary use and AI

**Fig. 9:**
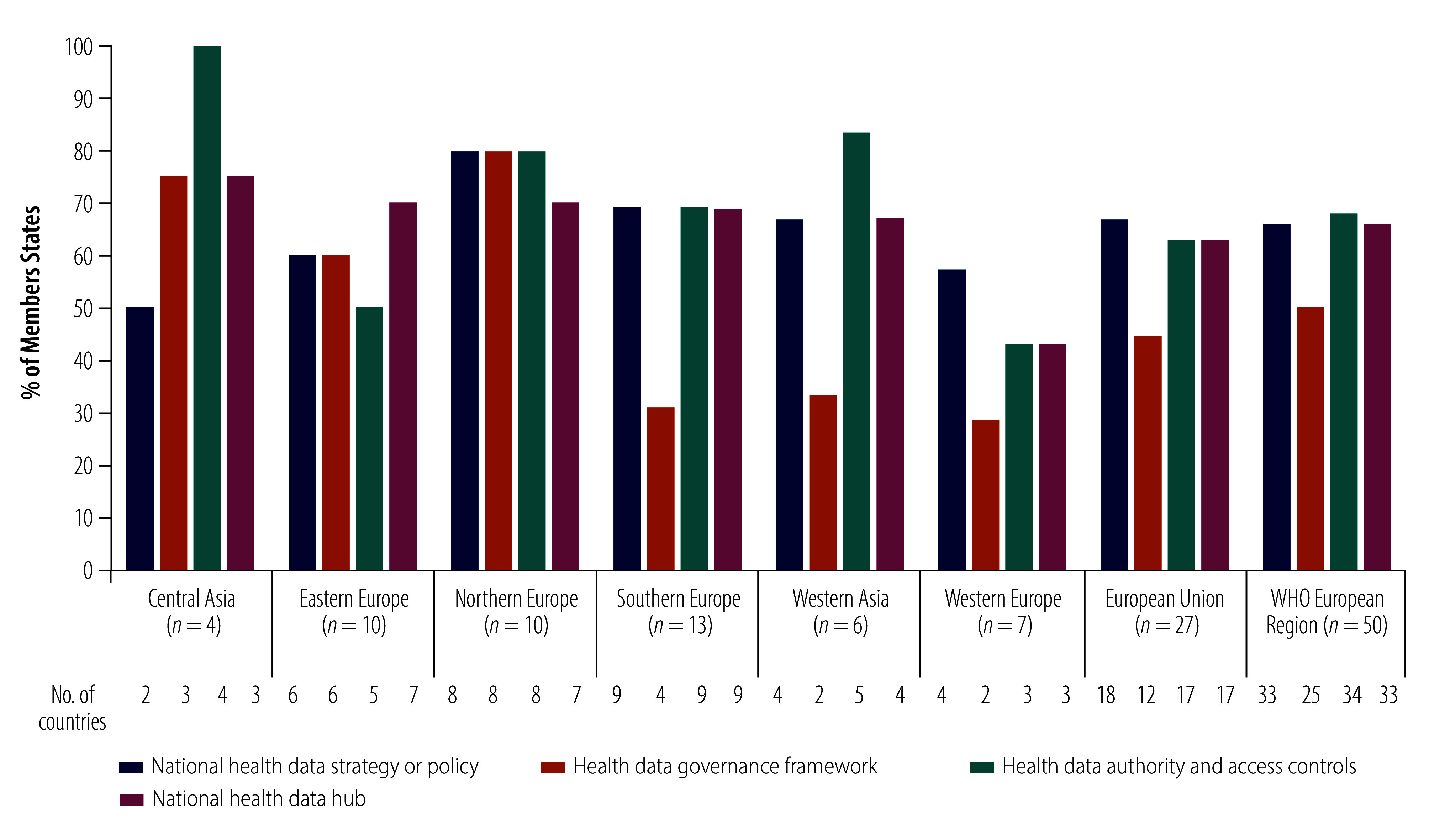
Institutions and measures in place for health data governance of artificial intelligence, by region and subregion, WHO European Region

Sixty-six percent (33/50) of Member States have a dedicated national health data strategy in place (Fig. 9), and 18% (9/50) have health data covered by their national data strategy or policy. Additionally, 66% (33/50) of Member States have created a health data hub at the subnational or national level. In regard to oversight, 68% (34/50) of Member States have set up a health data authority, which we defined in the survey as a body responsible for health data governance, the approval of requests for new data set creation, or data set access, linkages or extraction.

WHO: World Health Organization.

Note: of the 53 Member States in the Region, 50 responded to the survey.

**Fig. 10:**
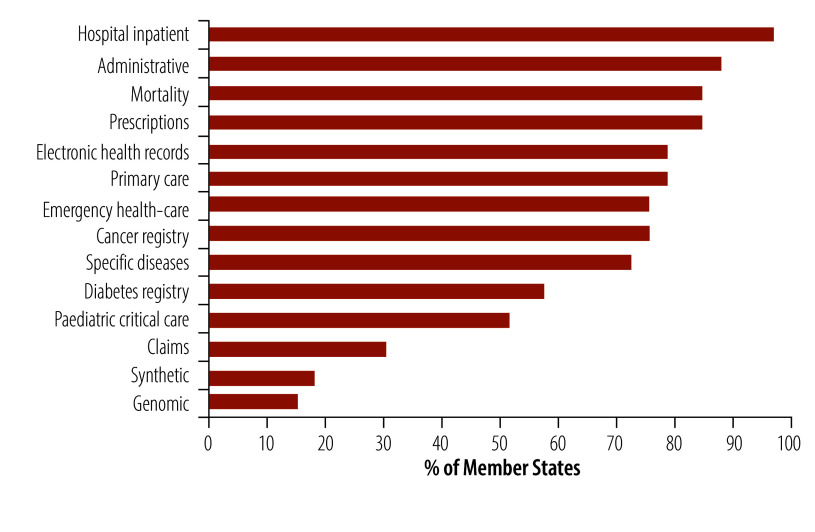
Type of data in health data hubs of Member States, WHO European Region

Of the Member States that reported having health data hubs, 97% (32/33) collected and stored inpatient data, while only 15% (5/33) collected genomic data (Fig. 10).

WHO: World Health Organization.

Note: graph only includes the 33 countries that reported having health data hubs.

In the EU, 44% (12/27) of Member States have adopted a health data governance framework.

Across subregions, adoptions of these frameworks remains low in western Europe (29%; 2/7) and southern Europe (31%; 4/13), while northern Europe shows the highest proportion with 80% (8/10) of Member States.

## Discussion

Member States have taken different approaches to AI strategy development. Some have chosen cross-sectoral frameworks and others have developed or are developing health-specific strategies. While health-specific AI strategies allow for tailored governance and faster implementation, they risk regulatory fragmentation without broader coordination. Conversely, cross-sectoral strategies can facilitate the establishment of unified standards, shared infrastructure and cost efficiencies, yet they may be less attuned to health-system priorities and slower to implement due to the need for coordination across multiple sectors. The reasons behind these strategy choices are largely unexplored in academic literature, highlighting the need for further research into the political, institutional and contextual factors that shape AI strategies for health care.

In the WHO European Region, responsibility for overseeing and implementing AI in health-care strategies typically rests with established governmental institutions. However, oversight faces several challenges including the rapid advancement of AI outpacing regulatory updates;^17^ expertise imbalances between large technology companies and public institutions; complicated evaluation of black box algorithms; and the influence of corporate lobbying and recruitment of former regulators.^3^

The legal and regulatory setting for AI in health across the WHO European Region, particularly within the EU, is complex and evolving. Member States are actively adapting and assessing their legal frameworks, developing new cross-sector legislation or amending existing laws to align with broader regulation oversight mechanisms. Our findings on the limited presence of new national health-specific AI laws align with other regulatory mapping.^18^ This mapping highlighted the developing and fragmented nature of dedicated AI regulation in health and the absence of binding national policies exclusively targeting AI in health care.^18^

The dynamic regulatory ecosystem for AI in health reflects a complex and interdependent governance model, wherein legal, ethical and operational dimensions are interconnected. For example, the EU AI act, a cross-sector regulation, serves as a foundational framework designed to operate in a broad regulatory ecosystem rather than in isolation.^7^ For a regulatory ecosystem to function effectively, cross-sector regulations, such as the EU’s General Data Protection Regulation, must operate in coordination with health-specific instruments including the Medical Devices Regulation, In Vitro Diagnostic Medical Devices Regulation and the proposed European Health Data Space.^14^^,^^18^ Similarly, national implementation of EU directives (e.g. the Open Data Directive and Cybersecurity Directive), along with additional national policies, such as specific health-data policies or national cybersecurity laws,^18^ should be designed to align with and reinforce these broader regulatory frameworks.

Ethical principles are increasingly embedded in the national AI strategies of Member States through regulatory mechanisms and oversight institutions, which is consistent with existing research.^3^^,^^19^ However, while abstract ethical guidelines are often seen as sufficient for steering the development of ethical AI, they frequently fall short in real-world implementation. Most frameworks emphasize what ethical AI should achieve but offer limited guidance on how to achieve it.^20^ Moreover, these ethical principles often serve as informal alternatives to regulation and lack enforceability, making compliance voluntary and inconsistent.^21^^,^^22^ In practice, corporate interests may override ethical considerations, reducing the influence of such principles and occasionally creating internal conflicts during the development and deployment of AI systems.^22^^,^^23^

In parallel, liability remains a challenge in the use of AI in health care, especially as clear legal standards exist in only a few Member States, limiting avenues for redress.^3^ Concerns include whether clinicians should be held responsible for following or ignoring AI recommendations that lead to harm, and how liability rules influence their reliance on such tools.^3^ Addressing these issues requires clearer legal frameworks and stronger institutional capacity to manage AI-related risks. 

Our findings show varying levels of policy maturity, investment capacity and data governance priorities across the WHO European Region, which concurs with other literature.^8^^,^^24^ The latest WHO digital health report shows strong privacy protections: all Member States have privacy laws and 91% (48/53) of Member States have legislation safeguarding health data in electronic health records.^12^ However, AI in health care challenges the principles of the General Data Protection Regulation such as data minimization transparency and individual rights. AI’s reliance on large, complex data sets often conflicts with the General Data Protection Regulation, especially regarding automated decisions and data subject rights.^25^

Data sharing and accessibility are central to AI governance and development, with health data hubs playing a main role in this process.^3^ Hospital and administrative data are most common due to their routine collection and structured formats. This finding aligns with WHO findings that electronic health records are used routinely in secondary care by 78% (38/49) of Member States and in tertiary care by 69% (33/48).^12^ In contrast, while 21 Member States have reported having infrastructure to facilitate the use of genomic medicine,^12^ our findings show that genomic data are the least available in health data hubs. This finding is likely due to the complexity of genomic data, ethical considerations and high costs. Technical barriers, such as lack of standardization and metadata interoperability, limit cross-study comparisons. Data files are often large and may need to be decrypted or converted to a standardized format, which slows down the process of data download and processing. These challenges make genomic data stewardship both important and complex.^26^

This study has several limitations. The main limitation of the survey is the relative flexibility of the definitions of terms used in the questions and the interpretation by each of the participating Member States. Furthermore, the validity of survey responses relied on the expertise of the national survey coordinator and the proficiency of the national experts in AI-related topics. Another limitation is that AI governance is rapidly evolving, meaning some findings may change in the near future. However, the survey’s strengths include its broad geographic and thematic coverage, the involvement of respondents within government, verification of responses using documentation, and the triangulation of responses across multiple stakeholders and informants, which enhanced the accuracy of the information collected.

The findings from this study suggest that several priority areas exist for policy action. First, national AI strategies should be developed and/or updated, whether health-specific or cross-sectoral, with a clear vision aligned to health priorities, time-bound goals, and robust monitoring and evaluation frameworks to ensure progress and accountability. Second, oversight needs to be institutionalized, and AI strategies implemented by assigning responsibility to established government agencies rather than temporary structures to ensure continuity, accountability and sustained execution. Third, liability frameworks should be established and clearly define responsibilities for developers, clinicians, data providers and institutions, with mechanisms for timely redress and accountability when AI systems cause harm. Fourth, ethical guidelines, such as WHO principles, need to be operationalized by embedding them into practical regulations and accountability mechanisms throughout the AI lifecycle. Fifth, robust data governance frameworks should be implemented that are aligned with international standards to protect individual rights, including informed consent, transparency and independent oversight. Health data hubs should maintain high standards with precise consent procedures, demonstrable public benefit and best-practice networks to support equitable implementation. Clear rules should distinguish public interest from commercial research and ensure transparency, benefit-sharing and protection of individual rights in collaborations. Guidance on secondary use and cross-border sharing of health data should enable secure, ethical and public-interest-driven research. These adaptive legal and policy actions are needed to respond effectively to the complex and evolving challenges of AI integration in health systems.
